# Purified Native Collagen Extracellular Matrix Plus Polyhexamethylene Biguanide Functions as a Barrier to Protect Complex Wounds in an In Vivo Model

**DOI:** 10.3390/ijms26189195

**Published:** 2025-09-20

**Authors:** Rami A. Nasrallah, Kelly A. Kimmerling, James L. Cook, Chantelle C. Bozynski, Aaron M. Stoker, James P. Stannard, Katie C. Mowry

**Affiliations:** 1 Organogenesis Innovation Center, San Diego, CA 92121, USA; 2 Organogenesis Discovery Center, Birmingham, AL 35243, USA; 3 Thompson Laboratory for Regenerative Orthopaedics, Missouri Orthopaedic Institute, Columbia, MO 65212, USA; 4 Department of Orthopaedic Surgery, University of Missouri, Columbia, MO 65212, USA

**Keywords:** polyhexamethylene biguanide (PHMB), complex wound, infection, methicillin-resistant *Staphylococcus aureus* (MRSA), purified native collagen extracellular matrix plus PHMB (PCMP)

## Abstract

Complex surgical wounds often necessitate repeated irrigation and debridement (I&D), resulting in substantial burdens. Purified native collagen extracellular matrix plus polyhexamethylene biguanide (PCMP) is a protective antimicrobial barrier that supports innate wound healing and was hypothesized to protect an in vivo complex wound. Canines underwent bilateral fibular defects, which were stabilized using screws and plates pre-incubated with methicillin-resistant *Staphylococcus aureus*. Wounds were clinically infected seven days post-op and underwent I&D prior to application with either PCMP or non-adherent pads. Outcome assessments included radiography, wound scoring, microbial culture, histology, and gene expression. PCMP application resulted in significantly improved clinical wound healing scores at days 3 and 7, while histological analysis trended towards improved wound repair. Radiological assessments showed no loosening, confirming implant stability. Quantitative microbial assessments showed a minor reduction in bacterial load (0.5 log fold-change) on day 10. Gene expression analysis showed significant upregulation of matrix metalloproteinases and immune-modulating cytokines. Protection of the wound with PCMP resulted in improved wound scores, reduced bacterial load, and significant upregulation in key gene expression pathways compared to controls. Overall, this study suggests that PCMP effectively limited bioburden and biofilm reformation and supported wound progression in a challenging environment. These findings suggest PCMP could enhance complex wound patient outcomes.

## 1. Introduction

An unsolved issue for management of orthopedic trauma and associated complex wounds involves the prevention and successful management of surgical site infections (SSIs). The Centers for Disease Control and Prevention (CDC) group SSIs into three categories: superficial incisional infections, deep incisional infections, and organ/space infections [1]. SSIs cause an enormous clinical burden on patients, hospitals, and payors. In 2014, Medicare spending estimates for surgical wounds, including infection costs, ranged from $11.7 billion to $38.3 billion, making up almost half of the total Medicare spending estimates for all wound types [2]. Additionally, patients with infections often require readmission to the hospital, resulting in further financial costs, loss of workdays, and a decrease in quality of life [1,3].

Traumatic and surgical wound infections start with microbial contamination, which can arise from the patient’s natural flora (endogenous) or the surrounding environment (exogenous) [1]. Regardless of the source of bacteria, risk factors for developing an SSI can involve patient factors, including age and comorbidities such as diabetes, obesity, nicotine use, or immune dysregulation [1,3,4,5]. Furthermore, lower extremity trauma, pre-operative skin preparation method, antimicrobial prophylaxis, breaks in aseptic technique, use of implants, duration of surgery, and time in the hospital can significantly influence SSI risk [1,4]. Importantly, the amount of time a wound remains open, especially in delayed procedures, significantly increases the risk of infection, and the SSI risk increases by approximately 1.3 times for every additional hour of surgery [6]. As such, efficient and effective wound management strategies are critical to the prevention and resolution of SSIs.

The two “gold standards” for the management of at-risk or infected surgical wounds are irrigation and debridement (I&D) and antibiotic therapy based on culture and sensitivity testing. While these two treatments can be effective at mitigating SSIs and promoting wound healing, I&D is often required every three to four days until the infection clears, and the injudicious or ineffective use of antibiotics risks antimicrobial resistance, which can make bacterial infections more difficult to resolve [3,7]. Furthermore, biofilm formation that is often associated with orthopedic implants in SSIs is highly resistant to many commonly used antibiotics and can reform rapidly following I&D [8]. The biofilm induces prolonged inflammation and extracellular matrix (ECM) degradation, leading to chronic SSIs and wound healing impediments that are extremely difficult to resolve [9,10].

Several antiseptic agents, such as silver, iodine, and polyhexamethylene biguanide (PHMB), have shown promise in reducing microbial activity and preventing biofilm reformation [11]. PHMB is a broad-spectrum antimicrobial agent that disrupts negatively charged microbial cell membranes through its positively charged cationic properties and further interferes with bacterial chromosomes [11,12]. It is reported to be effective against a wide range of pathogens, including Gram-positive and Gram-negative bacteria, methicillin-resistant *Staphylococcus aureus* (MRSA), fungi, and viruses [11]. Commercially, PHMB has been incorporated into different types of wound dressings, including collagens, gauzes, and foams [3,11]. One of these dressings is a porcine native cross-linked collagen matrix with PHMB (purified native collagen ECM plus PHMB; PCMP). PCMP is made from porcine small intestine submucosa (SIS), which is a natural ECM scaffold that can support cell attachment, migration, and proliferation [13,14]. Collagen matrices have also been reported to help support progression through the wound healing cascade [15]. Integrating PHMB into a native collagen matrix allows for antimicrobial effectiveness within PCMP to help prevent biofilm reformation and support effective wound healing. As such, PCMP has the potential to beneficially contribute to the management of at-risk surgical wounds.

To explore the use of PCMP in these complex wounds commonly associated with orthopedic trauma, a validated clinically relevant translational canine acute fracture-related infection model was used [16]. The purpose of this study was to evaluate whether PCMP would protect a complex wound environment for seven to ten days, potentially reducing the need for ongoing I&D. The hypothesis was that PCMP would help protect wounds from biofilm reformation after I&D and support the progression through the innate wound healing cascade compared to controls.

## 2. Results

### 2.1. Model Overview, Generation, and Study Timeline

**Figure 1:**
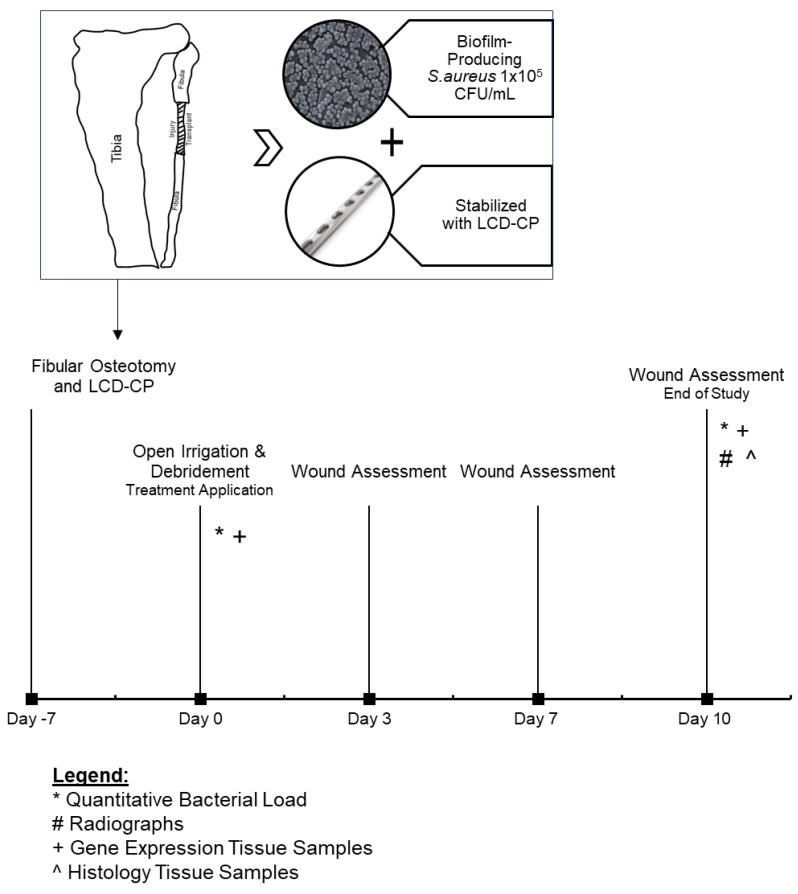
Canine model overview and study timeline. LC-DCP: limited contact dynamic compression plate; CFU: colony-forming unit.

An acute fracture-related infection (FRI) model in the fibula was successfully used to replicate complex orthopedic wounds and outcome measures were assessed (Figure 1).

**Figure 2:**
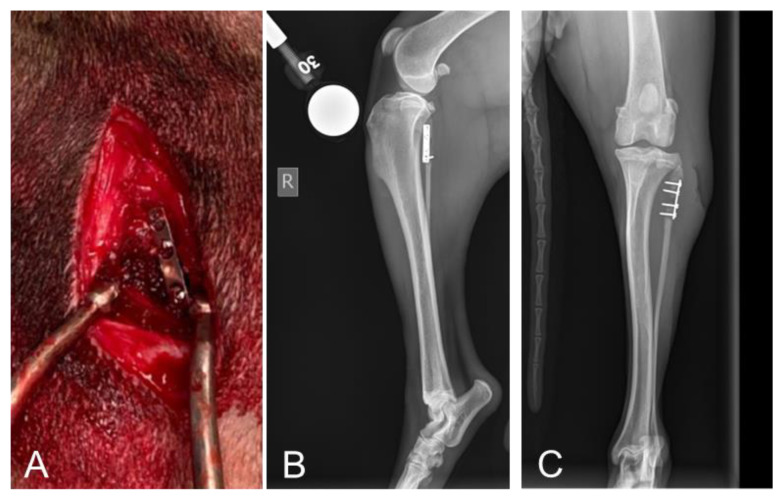
Surgical procedure images. (**A**) Intra-operative image of the exposed proximal fibula in a canine with the surgical placement of the limited contact dynamic compression plate (LC-DCP). (**B**) Representative radiograph of the stabilized fibular defect with a right lateral view. (**C**) Representative radiographs of the stabilized fibular defect with an anteroposterior view.

Representative images showing successful surgical placement of the methicillin-resistant *Staphylococcus aureus* (MRSA)-coated limited contact dynamic compression plate (LC-DCP), along with radiographic images, are shown in Figure 2.

Additional details about the study and outcome measures can be found in the Materials & Methods section.

All dogs underwent assigned treatments and survived for the intended duration of the study with retained implants and no evidence of unmanaged pain, pain-related dysfunction, or systemic infection. Daily participation in socialization and enrichment activities was documented for each dog.

### 2.2. Radiographic Assessments Revealed No Implant Failures Following 10 Days of Treatment

Subjective radiographic assessments were consistent among dogs with evidence of soft tissue swelling, implant-associated osteolysis, implant loosening, periosteal reaction, and non-bridging callus formation. Bone union was not achieved during the 17-day study period; however, implant failure did not occur (Figure 2). This substantiates the suitability of the preclinical canine fibular defect model with *S. aureus*-contaminated implants for accurately assessing complex wound healing associated with FRI-related surgical site infections (SSIs).

### 2.3. Clinical Assessments Showed Significantly Improved Wound Healing at 3 and 7 Days with Purified Native Collagen Extracellular Matrix Plus Polyhexamethylene Biguanide (PCMP)-Treated Wounds

**Figure 3:**
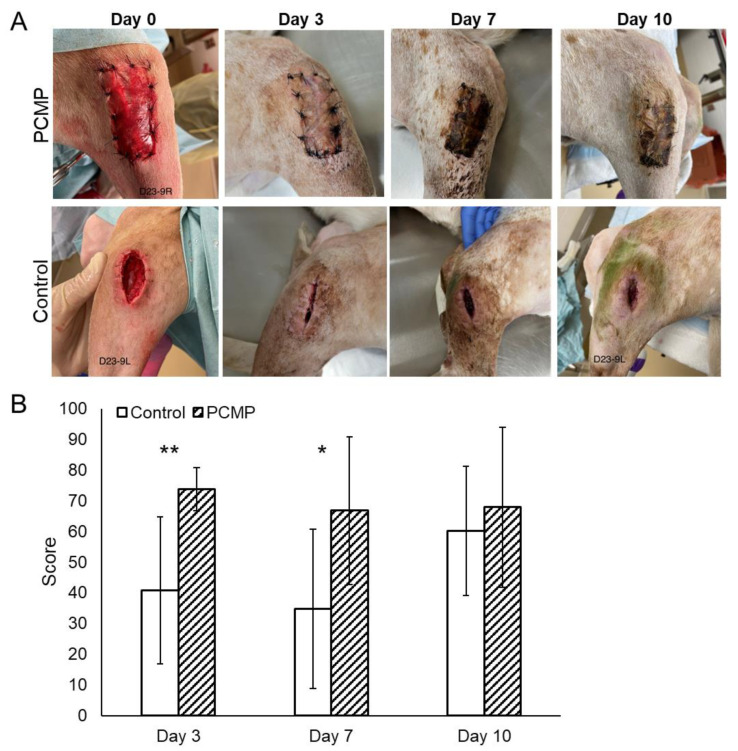
Representative gross wound images and wound assessment scores. (**A**) Representative wound images of porcine collagen matrix with PHMB (PCMP) and control groups at days 0, 3, 7, and 10 post-treatment. (**B**) Wound assessment scores for PCMP and control groups at days 3, 7, and 10 post-treatment. Scores range from 0 to 100, with 0 being extremely poor and 100 being excellent appearance of the wound. Mean ± standard deviation reported; *n* = 8 per group; * *p* < 0.05, ** *p* < 0.01. PHMB: polyhexamethylene biguanide; PCMP: purified native collagen extracellular matrix plus PHMB.

Before irrigation and debridement (I&D) (day 0), all surgical wounds exhibited clinical evidence of infection, including redness, swelling, edema, and mild to moderate serosanguinous or purulent drainage (Figure 3A).

However, all surgical incisions remained intact without wound dehiscence, and no signs of systemic infection were noted in any dog. On days 3 and 7, PCMP wounds were associated with significantly higher wound assessment scores than the control group (day 3: 73.8 ± 7 versus 40.8 ± 24, *p* = 0.001; day 7: 66.8 ± 24 versus 34.8 ± 26, *p* = 0.024), respectively (Figure 3B). By day 10, although PCMP wound scores trended higher compared to the control group (67.9 ± 26 versus 60.2 ± 21), the improvement did not reach statistical significance (*p* = 0.53).

### 2.4. Histological Assessments Showed Improved Wound Healing in PCMP-Treated Wounds

**Figure 4:**
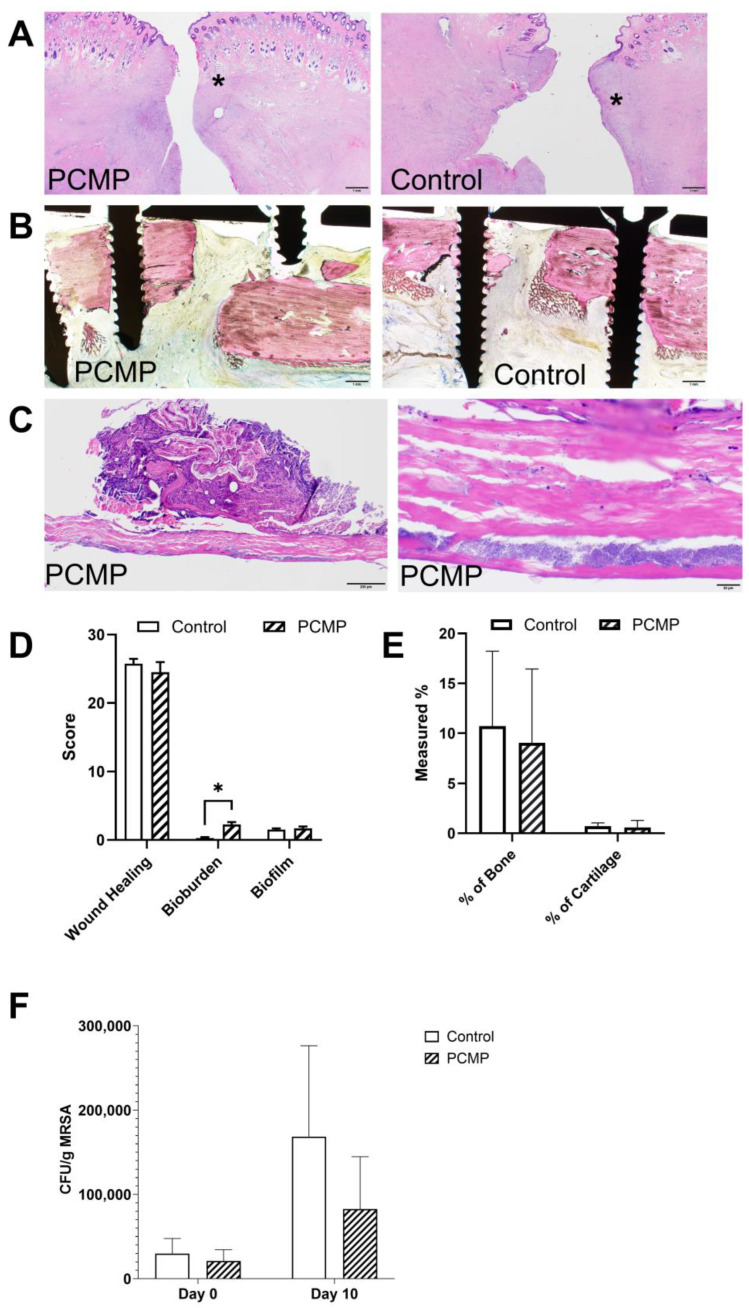
Representative photomicrographs of skin and fibula at surgical wound and fracture-repair sites. (**A**) Representative images showing wound healing at the surgical sites (denoted by *) for the porcine collagen matrix with PHMB (PCMP) and control groups at day 10 post-treatment. Scale bars indicate 1 mm. (**B**) Representative images showing fibrovascular tissue/granulation tissue within the fracture gaps (no callus formation) for the PCMP and control groups at day 10 post-treatment. Scale bars indicate 1 mm. (**C**) Representative low and high magnification images demonstrating bacterial infiltration in the PCMP matrix. Scale bars indicate 200 µm and 20 µm, respectively. (**D**) Histological assessment, including wound healing, bioburden, and biofilm. Mean ± standard deviation reported; *n* = 8 per group; * *p* < 0.05. (**E**) Histological assessment, including % of bone, and % of cartilage. Mean ± standard deviation reported; *n* = 8 per group. (**F**) Quantitative microbial load results showing the difference between day 0 (before irrigation and debridement, I&D) and day 10 post-treatment for the PCMP and control groups. Mean ± standard deviation reported; *n* = 7 per group. PHMB: polyhexamethylene biguanide; PCMP: purified native collagen extracellular matrix plus PHMB.

Histological assessments of all surgical wounds revealed a substantial open wound, as expected, with a defect spanning the epidermal, dermal, and subcutaneous layers, extending to the skeletal muscle level. The reparative tissue exhibited characteristics indicative of the proliferative phase of wound healing, including granulation tissue, fibrosis, hemorrhage, edema, and a mixture of inflammatory cells (Figure 4A). Histological assessments of the FRI sites revealed prominent fibrovascular tissue and granulation tissue within the fracture gaps, with variable quantities of mixed-cell inflammation, consistent with the expected level of bone healing at 10 days post-treatment (Figure 4B).

At the wound surfaces, retention of coccoid bacterial colonies was observed on and within the PCMP matrix. (Figure 4C). In contrast, the control group, where the non-adherent pad was replaced on days 3 and 7, exhibited minimal coccoid bacteria on the skin surface, resulting in a statistically significant difference in this subjective histologic bioburden score between the two groups (*p* = 0.016) (Figure 4D). This difference in superficial wound appearance is attributed to the PCMP being fixed to the wound bed throughout the study, whereas the non-adherent dressing was removed and replaced multiple times.

Upon evaluating the histologic cellular debris and cellular response scores, a trend was observed for the PCMP group to be associated with more favorable subjective wound healing scores compared to the control group (22 versus 26, respectively; lower scores indicate better histologic characteristics) (Figure 4D). Although these scores favored the PCMP group, the difference did not reach statistical significance (*p* = 0.291).

No statistically significant differences were noted between PCMP and control groups for the percentages of bone (*p* = 0.891) or cartilage (*p* = 0.210) in the ostectomy gaps or for the extent of biofilm associated with implants (*p* = 0.46) (Figure 4E).

### 2.5. Quantitative Bacterial Analysis Showed a Reduction in Bacterial Load in the PCMP Group After 10 Days

At the time of I&D, 12 out of 16 fibular fracture repair sites exhibited associated infections, with substantial growth of *S. aureus*. By the study endpoint (day 10), 15 out of 16 fracture-repair sites still had associated infections, showing abundant *S. aureus* growth in both the PCMP and control groups. However, when looking at the bacterial levels in each group at each time point, the PCMP group showed similar levels of bacteria at day 0 prior to treatment, and a slight reduction in bacterial load on day 10 compared to the control group (Figure 4F). Swab cultures collected from either the non-adherent dressing pad or PCMP on days 3 and 7 revealed the presence of *S. aureus* and various environmental bacterial contaminants at the wound sites, as shown on day 10 following histological assessment in Figure 4C.

### 2.6. Comparative Gene Expression Analysis Showed Significant Differences in Matrix Metalloproteinase (MMP) and Inflammatory Targets in PCMP-Treated Wounds

**Figure 5:**
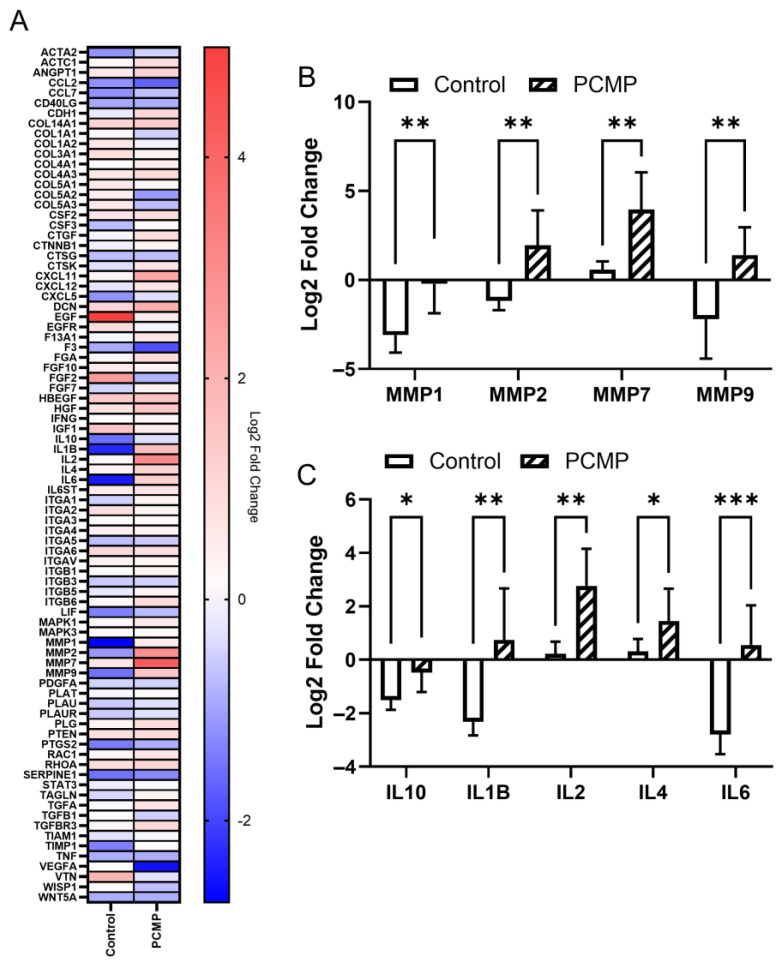
(**A**) Heat map summarizing gene expression changes in PCMP-treated wounds and control wounds of 84 wound healing-related genes. Gene expression changes in (**B**) matrix metalloproteinases (MMPs) and (**C**) immune-modulating cytokines. For all graphs, mean ± standard deviation reported; *n* = 7 per group; * *p* < 0.05, ** *p* < 0.01, *** *p* < 0.001. PCMP: purified native collagen extracellular matrix plus PHMB; PHMB: polyhexamethylene biguanide; MMP: matrix metalloproteinase; IL: interleukin.

To investigate the underlying mechanisms of wound healing and observed differences between the PCMP-treated and control groups, we compared gene expression profiles of full-thickness excisional wounds from both treatment groups at 10 days post-treatment relative to pre-treatment baseline (day 0). A summary heat map of the arrays targeting 84 genes related to wound healing is shown in Figure 5A, while a heat map with each individual animal can be found in Supplemental Figure S1.

Quantitative ribonucleic acid (RNA) expression analysis revealed significant differences in genes related to MMPs and inflammation pathways (Figure 5B,C). PCMP wounds exhibited an upregulation in the expression of genes associated with proteases, specifically MMP-1, MMP-2, MMP-7, and MMP-9 (*p* < 0.05 for all; Figure 5B). Furthermore, the PCMP wounds showed significantly higher expression of cytokines related to immune modulation, including interleukin-10 (IL-10), interleukin-1 beta (IL-1B), interleukin-2 (IL-2), interleukin-4 (IL-4), and interleukin-6 (IL-6) (*p* < 0.05 for all; Figure 5C) as compared to control.

## 3. Discussion

In this study, the use of a purified native collagen extracellular matrix plus polyhexamethylene biguanide (PCMP) protective antimicrobial barrier supported improved clinical wound healing scores during the first week of complex wound management associated with acute fracture-related surgical site infections. Additionally, there were no implant failures in the PCMP group. Histological assessments supported the differences in PCMP wounds compared to controls, as evidenced by increased granulation tissue, neovascularization, and cellular inflammation indicative of the proliferative phase of effective wound healing. A slight reduction in microbial load was seen following management with PCMP; however, the PCMP remained on the wound for the duration of the study. Gene expression analysis of tissue biopsies showed that PCMP-managed wounds had significant upregulation of matrix metalloproteinases (MMPs; MMP-1, -2, -7, -9) and immune-modulating cytokines (interleukins, IL; IL-1B, IL-2, IL-4, IL-6, IL-10), indicating that the protective barrier supported progression through the natural immune system in the wound bed.

The results of the present study further validated this preclinical canine model’s relevance for assessment of complex wounds associated with acute fracture-related infections (FRI) [16]. Importantly, FRIs are frequently associated with implant-associated biofilm formation and antibiotic-resistant surgical site infections (SSIs), resulting in significant morbidity requiring prolonged care, extensive healthcare costs, and undesirable outcomes [1,3,4,17]. Standard of care treatment includes multiple reoperations for irrigation and debridement (I&D) and implant exchange, as well as prolonged antibiotic treatment [3,7,18,19,20]. As such, novel methods for the prevention and resolution of these FRI-related SSIs are needed to improve patient outcomes and mitigate the burden on healthcare systems. Antimicrobial dressings are thought to provide a protective barrier to the wound by preventing bacteria from passing through the dressing itself. In a previous study, PCMP was compared to other collagen-based dressings in vitro, including an antimicrobial hydrofiber dressing, a collagen extracellular matrix, a collagen wound matrix, and scaffolds utilizing silver; PCMP had a significantly larger zone of inhibition compared to the antimicrobial hydrofiber dressing, collagen extracellular matrix, and collagen wound matrix at days 0–10 using methicillin-resistant *Staphylococcus aureus* (MRSA) [21], and its antimicrobial activity was sustained for a longer duration. Additionally, multiple studies have shown success with the use of collagen and bone matrices as scaffolds in tissue repair, including biodegradable collagen carriers with growth factors [22], absorbable collagen matrices [23], and coupled bone growth factor matrices [24]. Antimicrobials such as iodine and silver have been shown to reduce biofilm formation [25], but have some drawbacks, such as systemic adsorption and cytotoxicity, respectively [11]. The results from the current study show that the wounds treated with PCMP as an antimicrobial barrier supported progression through the wound healing cascade, as evidenced by clinical, histological, and gene expression data. Conversely, the standard-of-care dressing control wounds were not associated with these clinical and histological findings, and produced lower expression of immune-modulatory genes, suggesting a prolonged inflammatory phase of wound healing in this FRI-related SSI model.

The assessment of wounds in this study was performed by blinded healthcare professionals to evaluate clinical indicators, including dehiscence, gapping, draining/exudate/pus, inflammation/redness/swelling, and surrounding tissue appearance, which were used to help assess wound status and determine the need for further interventions [26]. The preclinical canine model used produces these same clinical indicators of ineffective wound healing and progression toward chronic FRI as noted in patients [27]. As such, the current study results demonstrate PCMP to be associated with significantly supporting wound healing; these results have a translational application in supporting a single application of a PCMP dressing combined with a single I&D and oral antibiotics for supporting progression of innate wound healing in the initial phases of acute FRI-related SSIs.

One advantage of using an animal model to study PCMP is the ability to perform gene expression analysis on tissue biopsies taken pre-treatment and 10 days post-treatment. Two categories of genes were primarily impacted on PCMP wounds: MMPs and immune-modulating cytokines. MMPs are involved in both wound healing and the management of infection through inflammatory cascades by the recruitment of inflammatory cells and cytokines to the wound site and have been shown to be induced by MRSA [28,29,30]. Dysregulated or overexpressed MMPs can cause wounds to remain in the inflammatory phase, resulting in chronic non-healing wounds [31,32]; however, increased levels of MMPs are required to resolve infection and proceed through the natural wound healing response [33]. In the current study, PCMP-treated wounds showed significant upregulation of MMP-1, -2, -7, and -9 compared to controls, suggesting that the inherent processes of infection clearance, immune response activation, and tissue remodeling were active [29]. PCMP-treated extremities also showed upregulated levels of IL-10, IL-1B, IL-2, IL-4, and IL-6. Increased levels of IL-2 and IL-4 are associated with T-cell-mediated adaptive immunity and correlate with increased Th1 and Th2 helper cells, respectively [34]. Th1 cells typically respond first by destroying infected cells, while Th2 helper cells act to clear infection and repair tissue [34,35]. Following this initial recruitment, IL-6 controls the switch to a regenerative environment, which is crucial to the progression of the innate wound healing response [36]. As part of this response, levels of IL-6 are elevated during the inflammatory and proliferative phase, and wane during the remodeling phase [36]. Additionally, elevated IL-1β levels are necessary for the body to mount its initial immune response; clinically, the lack of an initial IL-1β response resulted in worse outcomes [27]. In the current study, the ratio of IL-1β:IL-10 in the PCMP group was elevated compared to the control (~6× higher); these findings correlate with a porcine study looking at MRSA infections in deep dermal wounds, where IL-1β was elevated, indicating an acute inflammatory response as part of the wound healing cascade on PCMP wounds, which was associated with supporting improved healing outcomes [37]. Taken together, the upregulation of MMPs in the PCMP-treated extremities provides evidence of infection clearance, including the recruitment of immune cells by MMP-1, -2, -7, and -9, the activation of defensins to kill MRSA by MMP-7, and the dampening of excess MMPs through the upregulation of IL-4 and IL-10 [28,38].

There were some limitations to this study. First, PCMP is a 2-layer product, which showed signs of degradation by day 10. In the future, the use of a thicker version of PCMP could increase the durability of the barrier, potentially allowing for longer time periods of wound protection; however, protection with PCMP was shown through day 7. Second, a short-term animal model was used in this study to mimic complications associated with complex orthopedic wounds [16,39,40]; the use of a longer duration study could yield additional insights but may raise ethical concerns. Ethical and pragmatic experimental design did not allow for the conclusion of additional controls, including the use of a non-adherent dressing pad, multiple applications of PCMP, or the use of an ECM scaffold without an antimicrobial. Further, multiple I&Ds were not included in the study design based on the desire to evaluate the potential for PCMP to support the minimization of reoperations without potentiating SSIs or delaying wound healing. Finally, a longer study duration with multiple assessments and terminal endpoints was not considered ethically or financially feasible and was unnecessary to assess the clinically relevant indication of initial management of acute FRI-related complex wounds.

## 4. Materials and Methods

### 4.1. Animal Model Design and Justification

With approval from the Institutional Animal Care and Use Committee (#16680), skeletally mature, purpose-bred female research hounds (*n* = 8; 1–2 years, 19.1–29.0 kg) were included in the study. Each dog underwent a comprehensive orthopedic examination by a board-certified veterinarian, including gait observation, to ensure the absence of any pre-existing pain or pathology. Throughout the study period, the dogs were individually housed in 25-square-foot runs, with daily monitored social interactions and access to enrichment items. A licensed Doctor of Veterinary Medicine (DVM) or a Registered Veterinary Technician (RVT) with advanced training in laboratory animal medicine evaluated the dogs daily to assess their general health, pain, attitude, appetite, and activity level.

Function was evaluated through visual examination of gait using a 10 cm visual analog scale (VAS) to assess weight-bearing, stance time, stride length, head movement, and load distribution. Pain was evaluated based on responses associated with canine pain (e.g., muscle tensing, resisting, flinching, yelping, turning to look or bite), with the observer recording pain levels using a dynamic (during movement) and interactive (during palpation of surgical sites) VAS. When the VAS pain score exceeded 2, tramadol and carprofen were administered as described below until documented resolution (VAS pain score < 2). Water and food consumption were recorded, and participation in daily social interaction and enrichment activities was monitored and recorded.

A fibular acute fracture-related infection (FRI) model was used to mimic complications associated with orthopedic wounds (Figure 1), allowing for a controlled investigation of treatments in a complex wound healing setting [16,39,40]. This model consistently produces clinical signs of local infection, delayed union with osteomyelitis, implant loosening, and biofilm formation on fracture-stabilization implants, closely mimicking the clinical scenario of surgical site infection (SSI). In this study, a 10-day follow-up period was chosen based on the typical expected duration of interventional management of complex wounds associated with FRIs. Importantly, the use of bilateral, stabilized 1 cm fibular defects in this model adheres to ethical considerations in animal research, following the principles of the 3Rs (Replacement, Reduction, and Refinement). This approach minimizes the number of animals used while still generating statistically robust data. Bilateral defects reduce the number of animals required, as each animal can serve as its own internal control, while addressing pain and dysfunction by preserving the tibia, stabilizing the fibula, and employing validated pain assessments and effective analgesics [16].

### 4.2. Surgical Procedures

To create the FRI model (Day-7), the dogs were premedicated with dexmedetomidine (5–10 μg/kg IV) and morphine (0.5 mg/kg IM), anesthetized with propofol (4–8 mg/kg IV) and isoflurane (1–4% inhaled in O_2_), and placed in dorsal recumbency to prepare both hind limbs for aseptic surgery (*n* = 16). Perioperative antibiotics (cefazolin 22 mg/kg IV) were also administered. For this surgery, the first treatment cohort and first limb were determined by individual coin flips. Through a posterior(caudo)-lateral approach, a 4 cm incision was made, and a 1 cm segment of each proximal fibula was osteotomized using a sagittal saw. After the ostectomy, each fibula was stabilized with a limited contact dynamic compression plate (LC-DCP) with cortical screws (DePuy Synthes, Raynham, MA, USA), which had been pre-incubated with a suspension of 1 × 10^5^ CFU/mL of a biofilm-producing methicillin-resistant *Staphylococcus aureus* (MRSA) isolate strain OJ1 (ATCC, Manassas, VA, USA). Routine closures of the fascial, subcutaneous, and subcuticular layers were performed using sutures, and the skin was closed with staples. The surgical placement of the LC-DCP is shown in Figure 2A, with orthogonal radiographic views shown in Figure 2B,C.

### 4.3. Post-Surgical Monitoring

Dogs were recovered from anesthesia (atipamezole HCl IM; same volume as dexmedetomidine), monitored daily for general health with assessment and documentation of pain and function, and provided analgesics (carprofen (4.4 mg/kg SQ) and 2 doses of morphine (0.5 mg/kg IM) given within 6 h of the preceding dose initially followed by tramadol (2–7 mg/kg PO) every 12 h for 3 days and carprofen (4.4 mg/kg PO) once a day for 7 days and then as indicated based on documented assessments of pain). Soft padded bandages were placed on both hindlimbs and replaced daily for 7 days.

### 4.4. Wound Management Cohorts

Seven days after model induction (Day 0), the dogs were premedicated, anesthetized, and prepared for aseptic surgery of both hind limbs, as previously described. The surgical sites, all of which produced drainage and showed clinical signs consistent with infection, were re-exposed via staple/suture removal from the previous incisions. Grossly devitalized and/or infected tissue was debrided using a scalpel, scissors, and curettes, followed by thorough irrigation (1 L of 0.9% saline) using pulsed lavage (Stryker InterPulse, Portage, MI, USA). The LC-DCP was left intact, and I&D was completed down to the bone.

Following the irrigation and debridement (I&D), deep fascial closure was completed using 2-0 PDS sutures, while the subcutaneous tissues and skin were managed as open wounds. Subsequently, either (1) PCMP was sutured into place with an overlying standard of care dressing (3M™ Tegaderm™ with non-adherent pad, 3500 Series, 3M, Saint Paul, MN, USA), or (2) only a standard of care dressing (3M™ Tegaderm™ with non-adherent pad; control) was applied to the open wounds, alternating between left and right hind limbs to minimize bias (*n* = 8/treatment cohort). PCMP is a commercially available porcine cross-linked extracellular matrix scaffold that consists of a collagen sheet coated with PHMB and sterilized using gamma irradiation (PuraPly^®^AM, Organogenesis, Canton, MA, USA). Sterile self-adherent cohesive wrap bandages and neoprene sleeves (Coodeo Dog Recovery Sleeves, Zhuhai, Guangdong, China) were placed on both hind limbs to protect the surgical sites. The self-adherent cohesive wrap bandages and neoprene sleeves were replaced daily, while the standard of care dressing was replaced as needed. The dogs recovered from anesthesia, were monitored daily for general health, with pain and function assessments documented, and provided with analgesics as previously described. Systemic antibiotics were administered to all dogs twice daily for 10 days (Trimethoprim-Sulfamethoxazole (TMS) 15–30 mg/kg PO).

### 4.5. Outcome Measures

#### 4.5.1. Radiographic Evaluation

After 10 days of treatment, anterior–posterior (craniocaudal) and mediolateral radiographic views of the hind limbs were obtained on Day 17. Radiographs were assessed by a board-certified veterinary radiologist, who was blinded to treatment type, to subjectively characterize fibula bone, implants, and associated soft tissues for clinically relevant diagnostic imaging characteristics.

#### 4.5.2. Wound Assessment Scoring

Wound healing was assessed at baseline (Day 0) and on Days 3, 7, and 10 post-treatment (post dressing application) using a wound scoring system based on suggestive (e.g., local redness or fever, new onset wound drainage) and/or confirmatory (e.g., fistula, sinus tract, wound breakdown, purulent drainage, and/or presence of pus) signs of infection [41]. Wounds were photographed by a laboratory technician who was blinded to treatment. Deidentified and randomly numbered photographs were independently assessed by three orthopedic surgeons blinded to treatment and scored over a range from 0 to 100, with higher scores representing better post-operative wound appearance based on dehiscence, gapping, draining/exudate/pus, inflammation/redness/swelling, and surrounding tissue appearance.

#### 4.5.3. Histological Assessments

Following humane euthanasia at Day 10 post-treatment, using pentobarbital sodium (Fatal Plus 390 mg/mL, Vortech Pharmaceuticals, Ltd., Dearborn, MI, USA; 1–2 mL/5 kg IV), tissues were collected for processing as undecalcified specimens (including plates and screws) embedded in methyl methacrylate and longitudinally sectioned through the approximate center of the plate and stained with Stevenel’s blue/van Gieson stain by Inotiv, Inc. (Everett, WA, USA). Collected skin wound specimens were embedded in paraffin and stained with hematoxylin and eosin (H&E) and Gram stain by Inotiv, Inc. (Everett, WA, USA); PCMP remained intact on applicable specimens. Two board-certified veterinary pathologists, who were blinded to treatment type, evaluated bone and wound specimens for callus formation/maturity, wound healing, biofilm presence/extent, and bacterial load. Clemex Vision PE software version 8.0 (Clemex Technologies Inc., Longueuil, QC, Canada) was utilized to measure the percentages of bone, cartilage, and other tissues at the FRI site.

#### 4.5.4. Microbial Culture

Microbial evaluation of each wound was conducted using a non-quantitative swab culture from treatment sites (intact PCMP or non-adherent dressing pad) on Days 3 and 7 post-treatment. In addition, a quantitative bacterial load assessment was performed using tissue cultures from specimens collected on the day of I&D and 10 days post-treatment.

Tissue specimens were collected from the fracture site/implant by excising a small tissue sample with a sterile scalpel. Microbial testing was performed by a veterinary medical diagnostic laboratory. For the isolation of aerobic and anaerobic bacteria, organisms were cultured on standardized media and incubated under standard conditions for aerobic, capnophilic, microaerophilic, or anaerobic bacteria. Colony counts were performed, and colony-forming units (CFU) per gram of tissue were calculated. Organisms were then identified using matrix-assisted laser desorption/ionization-time of flight (MALDI-TOF) mass spectrometry, biochemical tests, and/or 16S deoxyribonucleic acid (DNA) sequencing.

#### 4.5.5. Gene Expression Analysis

Tissue specimens were collected from the wound site using a sterile scalpel at day 0 (pre-treatment) and day 10 (post-treatment) and processed for gene expression analysis to assess the impact of treating wounds with PCMP compared to baseline levels on day 0. Tissue samples were immediately transferred into RNAlater (AM7021, Life Technologies, Carlsbad, CA USA) storage solution, stored at −80 °C, and thawed on ice prior to processing. Total ribonucleic acid (RNA) extraction was completed using the RNeasy Fibrous Tissue Mini Kit (74704, Qiagen, Hilden, Germany) per the manufacturer’s protocol. Briefly, tissue was homogenized with a TissueRuptor II rotor-stator homogenizer in tissue lysis buffer (RLT Buffer, Qiagen, Hilden, Germany). RNA concentration was measured using a NanoDrop ND-1000 (NanoDrop Technologies, Inc., Wilmington, DE, USA) with absorbance at 260 nm. Purity was evaluated by the A260/A280 ratio, which is required to be between 1.8 and 2.0.

For real-time quantitative polymerase chain reaction (RT-qPCR) assays, RT^2^ SYBR^®^ Green qPCR Master-Mix (330513, Qiagen, Hilden, Germany) was used following cDNA synthesis with the RT^2^ First Strand Kit (330401, Qiagen, Hilden, Germany). Quantitative RNA expression of 84 genes was performed using RT^2^ Profiler PCR Dog Wound Healing Arrays (330231, Qiagen, Hilden, Germany) using an Applied Biosystems 7500 Real-Time PCR System (Applied Biosystems, Foster City, CA, USA). Standard ∆Ct and fold-change (2^−ΔΔCt^) of normalized gene expression were calculated using the GeneGlobe Data Analysis Center (Qiagen, Germany). Outlier analysis using ROUT with Q = 1% and log transformation was performed in GraphPad Prism (GraphPad Software, La Jolla, CA, USA). Differential gene expression was measured on day 10 post-treatment and normalized to baseline levels of skin following I&D at day 0.

### 4.6. Statistical Analyses

Means, medians, and standard deviations for wound scores and histomorphometry percentages were calculated. Statistical analyses were conducted using GraphPad Prism 10 software to compare the PCMP and control groups, employing paired sample *t*-tests for continuous data and rank-sum tests for categorical data. Significance was denoted as * *p* < 0.05, ** *p* < 0.01, and *** *p* < 0.001.

A power analysis was performed prior to this study; using data from previous wound scoring studies, a *t*-test calculation based on an expected mean difference of 25 and standard deviation of 15 determined that a sample size of 8 was sufficient to achieve a power of 0.8 with alpha = 0.05.

## 5. Conclusions

In conclusion, in a fracture-related infection model, a single application of PCMP dressing combined with a single I&D and oral antibiotics supported progression of wounds through the innate wound healing cascade when compared to standard-of-care dressings. Based on clinical, histological, and gene expression data, PCMP acted as an antimicrobial barrier and scaffold, limiting bacterial infiltration. This antimicrobial effectiveness within PCMP helped to manage bioburden and biofilm reformation. Future studies evaluating thicker versions of PCMP may help extend treatment periods in complex orthopedic wounds using this preclinical canine model; however, this study provides evidence that PCMP may be beneficial for use in the initial management of orthopedic trauma-related complex wounds with potential to improve outcomes for patients.

## Data Availability

The data that support the findings of this study are available from the corresponding author upon reasonable request.

## Supplementary Materials

The following supporting information can be downloaded at: https://www.mdpi.com/article/10.3390/ijms26189195/s1.
