# Supplementary material for: Purified Native Collagen Extracellular Matrix Plus Polyhexamethylene Biguanide Functions as a Barrier to Protect Complex Wounds in an In Vivo Model
Source: Int J Mol Sci. 2025 Sep 20;26(18):9195. doi: 10.3390/ijms26189195 (PMC12470437; doi:10.3390/ijms26189195)
Supplement: Supplementary file 1 [file ijms-26-09195-s001.zip › ijms-3846137-supplementary.pdf]

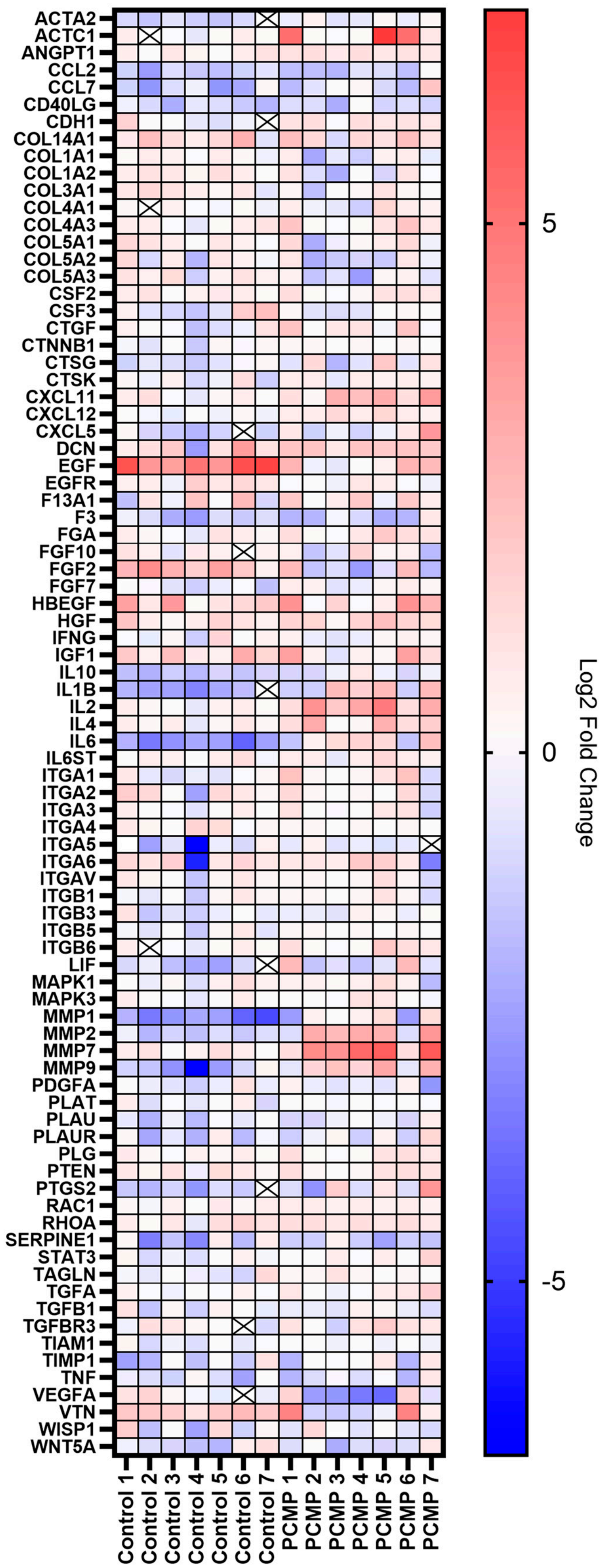

Figure S1: Heat map of individual animal gene expression changes in PCMP-treated wounds and control wounds of 84 wound healing-related genes. Outlier analysis (ROUT) was performed, and outliers indicated as X were removed
